# Subacute Pneumatic Arthritis

**Published:** 1877-05

**Authors:** Erenæus S. Davis

**Affiliations:** Allentown, Pa.


Subacute Rheumatic Arthritis.—*By Erenæus S. Davis, M.D., of Allentown, Pa.*—In the *Reporter* of January 6th, a correspondent asks for suggestions in the treatment of rheumatic arthritis. It is much as if one should ask for a plan for the cure of fever. The conditions liable to be called rheumatic arthritis, and which, by different persons, are severally recognized in that name, are quite various, and of every degree of gravity, from neuralgia affecting the capsule and surrounding parts, to general acute rheumatism. It is, indeed, rather rare to find two physicians of ordinary intelligence and experience, who will fully agree in their nosology, or even in their diagnosis and etiology of diseases commonly comprehended in this class. So much confusion exists in this regard, that it could almost be wished that the very name of rheumatic arthritis might be totally abandoned, and a more exact nosology adopted. It is not always easy to discriminate between some of these conditions, although they are essentially different. The case referred to is most probably what, from analogy and for convenience, we may call subacute rheumatic arthritis. This is quite a different affair from either the acute or the chronic form. It rarely or never occurs except where there is a pre-existing rheumatic diathesis. It is gradual in its accession, usually giving little trouble for months, or even years, and the first few years of its existence frequently comprise considerable periods of entire remission; except when complicated with neuralgia or some form of malarial disease, it is but moderately affected by meteorological conditions. It affects, by preference, the wrists and ankles, and is more likely to extend toward the extremities than in the opposite direction. Frequently it is confined to a single joint. It is commonly first noticed as a slight lameness, or perhaps, only a sense of weakness, and is likely to be attributed to a supposed sprain. A careful examination at this time will detect a very slight tenderness of the articular cartilage, with, possibly, a corresponding tenderness of some of the ligaments, but nothing more. It is to be distinguished from simple arthritis, or synovitis, by the absence of effusion within the capsule, from chronic rheumatic arthritis by the free mobility of the joint, the absence or small degree of infiltration, and the history of the case, and from a sprain by the absence of febrile symptoms, of discoloration, of extensive infiltration, and of any considerable localized tenderness in the ligaments. It is not likely to be mistaken for acute rheumatic arthritis. As time passes, the symptoms become intensified, and frequently there is very considerable febrile action around the joint, accompanied by a moderate amount of infiltration, though from first to last there is little or no effusion within the capsule. At this stage the pain is constant, but subject to great exascerbations. Usually, there is more pain toward the latter part of the day, or in the evening, as well as when general fatigue has been incurred. The skin often now becomes red, shining, and exquisitely tender, and sometimes, from reflex irritation, the muscles, both flexors and extensors, become permanently contracted. From this cause, and also from the pain which motion now produces, the joint at length becomes rigid, and may be quite anchylosed

The treatment must aim to remove or abate the systemic vice which gives rise to the disease. Gradually, and with much hesitation, I have been led to look upon the iodide of iron as almost a specific for this purpose. The form in which I prefer to use it in these cases is in pills, made according to the formula of the United States Pharmacopœia for 1851, as follows:—

| | | |
|---|---|---|
| ℞. | Ferri sulphat, . . . . . . | ʒj |
| | Potass iodid., . . . . . . . | ℈iv |
| | Tragacanth pulv., . . . . . | gr.x |
| | Sacch. pulv., . . . . . . . | ʒss |
| | Syrup, . . . . . . . . . | qs M. |

Ft. mass., et in pil. No. xl div.

Two or three of these pills may be given after each meal. The iodide of potash has not, with me, proved nearly so efficacious in these cases as this preparation, nor have other forms of the iodide of iron. I always have the pills freshly prepared when wanted, and whether it is owing to this fact, or to the presence of sulphate of potash with iodide of iron, resulting from the double decomposition, or to some other cause, I cannot tell, but I have rarely met with a case of subacute rheumatic arthritis which was not arrested by this treatment if used in the early stages. Later in the course of the disease the prognosis is not so hopeful, but still the same general plan of treatment is indicated, while it should be supplemented by measures addressed to special conditions. Most of the phenomena which are developed late in the history of the disease are really complications depending wholly or in part on reflex irritation, or on some inter-current cachexia or diathesis. Malaria and various neuroses are among the most common complications. All these are to be carefully searched out and appropriately treated. Neglected, they seriously aggravate the original disorder, and render a cure almost impossible. The inflammation of the skin and the infiltration are usually of reflex or of neurotic origin. Many of the complicating neuroses are well treated by electricity, though it is at least doubtful whether this ever has any directly beneficial effect upon the disease itself. Occasionally surgical interference may become a necessary auxiliary to the general treatment, as, for example, in some cases of rigidity or anchylosis, great muscular contraction, or excessive superficial inflammation. When the pain is very troublesome the following liniment will often be found to give great relief:—

℞. Tinct. iod.,

Glycerin, . . . . . . aa. ℥j

Linim. sapon. camph., . . . ℥ij M.

It is to be borne in mind that nearly every case of this affection is anæmic. Frequently they have the appearance of plethora, but a close observation will almost invariably prove that this appearance is generally caused by a fatty deposit, by no means indicative of a healthy state of the blood.

Another thing to be remembered is, that, though the disease be cured, the diathesis remains, and a new manifestation may occur at any time. The best prophylactic management consists in maintaining the highest possible condition of general health.—*Med. and Surg. Reporter.*

